# NEWS

**DOI:** 10.7189/jogh.03010203

**Published:** 2013-06

## DEMOGRAPHY

• According to a study released in 2012 by the Pew Research Center, the birth rate in the USA is the lowest in nearly a century. This trend is a global phenomenon, with women in most places today having fewer kids, and later in life. In 1970 the average woman on the planet gave birth to 4.7 children in her lifetime. By 2011 that number had dropped to 2.5. Even in the world’s most fecund region, sub–Saharan Africa, the fertility rate fell from 6.7 to 4.9 between 1980 and 2010, with births among women under 20 dropping by 20% in the 21st century. The combination of falling birth rates and longer life expectancies also means the world is rapidly getting older. In 1970, the median age was 20; in 1980, it rose to 23; by 2050, it will be 38. Meanwhile, the number of people older than 65 years doubled from 5% to nearly 10% between 1970 and 2011 and is expected to further rise to 20% by 2050. Expenditures on the old will skyrocket, with pension spending in the European Union already reaching about 12.5% of GDP. (*BusinessWeek*, 7 Feb 2013)

• Antiretroviral (ARV) drugs for HIV have had a profound effect on life expectancy in Africa, a study in the *Science* magazine showed. Adult life expectancy had fallen to only 49 years in KwaZulu–Natal, South Africa, in the year 2003, and widespread use of antiretroviral drugs was introduced in the fall of 2004. From that point, life expectancy kept increasing and reached 60.5 years by 2011. Adult life expectancy is the mean age to which a 15–year–old could expect to live if subjected to the full pattern of age–specific mortality rates observed in a population for a given period of time. (*BMJ*, 21 Feb 2013)

• UNICEF's report “Generation 2025 and beyond: the critical importance of understanding demographic trends for children of the 21st century” highlighted that, by the middle of the 21st century, almost one in every three births will be in sub–Saharan Africa. In comparison, this region had just one in ten births in 1950. The main factors contributing to this scenario will be improved child survival and continuing high fertility rates in sub–Saharan Africa, which contrasts with the falling rates in the rest of the world. In 1990, about half the world's children lived in developing countries while by 2025 nearly two–thirds will live in these countries (*The Lancet*, 2 Mar 2013).

• One of the most surprising, and perhaps confounding, facts of charity in America is that the poorest people donate the greatest percentage of their income. In 2011, the Americans whose earnings were in the top 20% of the population contributed 1.3% of their income to charity, while those in the bottom 20% donated 3.2%. This difference is further accentuated by the fact that, unlike middle–class and wealthy donors, most of them cannot take advantage of the charitable tax deduction. Some experts now speculate that the wealthy may simply be less generous, because the personal drive to accumulate wealth is inconsistent with the idea of communal support. Dr Paul Piff, a psychologist at UC Berkeley, conducted research that correlated wealth with an increase in unethical behaviour, saying that “...the rich are more likely to prioritize their own self–interests above the interests of other people.” (*The Atlantic*, 20 Mar 2013)

• In their annual progress report on the MDGs, the World Bank and the International Monetary Fund (IMF) focused on the positive effects of urbanisation. Urbanisation could be a “force for good”, providing better jobs and cheaper services and accelerating progress towards the Millennium Development Goals (MDGs). However, careful planning is essential to prevent the growth of slums, pollution and crime, all of which could prevent reaching the agreed targets. The report stated that urbanisation provides higher incomes than workers would earn on a farm and yields many opportunities to climb the income ladder. (*The Guardian*, 17 Apr 2013)

## ECONOMY

• The United Nations' report in December 2012 presented a grim economic forecast, described a cycle of austerity, and exposed persistent unemployment in many countries. The triple threat of the ‘fiscal cliff’ in the United States, the European debt crisis and the economic hard landing in China spared little optimism at the United Nations headquarters. The report’s main author, Mr Robert Vos, who is the Director of the Development Policy and Analysis Division of the United Nations Department of Economic and Social Affairs, feared the exacerbation of this economic climate could cause “a new global recession”. If this was indeed the case, then the unemployment lost across the United States and Europe since 2008/2009 would be unlikely to recover until as late as 2017. Although the presented proposals on the best strategies to avoid this outcome will not be easily incorporated into the tight budgets of the United States and Europe, it has still provided one of the most comprehensive assessments of the world’s economic landscape and exposed what United Nations experts view as the most pressing areas of concern. (*New York Times*, 18 Dec 2012)

• In his debut speech to the UN General Assembly, French President Mr Hollande asked attending world leaders to “globalize solidarity” and implement a Financial Transaction Tax (FTT). In a pioneering move, he announced part of the new French FTT will be allocated to development aid; if other nations choose to follow, this move could offer a solution to plugging holes in foreign aid which has been drying up throughout the current economic crisis. There is already evidence that globalization can give back to the global poor using small levies on transactions: UNITAID. This global health organization raised over US$ 2 billion over the last six years through a small levy on air tickets in nine countries including France, Chile and South Korea. This secure line of funding is then used to negotiate the best prices for tests and treatment for HIV/AIDS, malaria and tuberculosis. France has demonstrated that FTT is feasible. (*The Huffington Post*, 10 Jan 2013)

• The World Bank has published its latest report showing some optimism for growth in the economy, however sluggish this might be. This is largely down to developing countries, which clearly have an accelerating pace of growth. These countries are also driving global increase in goods and service trade, one of the key successes described in the report. However, the report also describes the need for these countries to modify their policy–making away from short–term fixes to counteract the developed world’s economical fluctuation, and to focus instead on addressing their domestic economic issues in order to stimulate long–term growth. The chief economist, Mr Kaushik Basu, stated that these countries should strive to “ensure their fiscal and monetary policies are robust and responsive to domestic conditions”. (*New York Times*, 15 Jan 2013)

• The report, by Oxford University's poverty and human development initiative showed that poverty is shrinking rapidly across the globe. Their study has taken a new approach to measuring deprivation. It predicts that countries among the most impoverished in the world could see acute poverty eradicated within 20 years, if they continue to progress at present rates. It identified “star performer” nations, such as Rwanda, Nepal and Bangladesh, where it predicts that deprivation could disappear within the lifetime of present generations. Following them closely in rapid reduction in poverty levels are Ghana, Tanzania, Cambodia and Bolivia. (*The Guardian*, 17 Mar 2013)

• The World Bank's president, Mr Jim Yong Kim, claimed that the recent signs of recovery in the global economy meant that there was now an “opportunity to create a world free from the stain of poverty” by 2030, although the optimistic new target has already divided development experts. The president of the World Bank has warned that ending the extreme poverty within a generation would be more difficult than tackling the AIDS pandemic and he urged direct action to help more than a billion people benefit from growth. He added that the goal of reducing the number of people living on less than US$ 1.25 a day from 21% to 3% by 2030 was achievable, although “extraordinarily difficult”. (*The Guardian*, 4 Apr 2013)

## ENERGY

• According to this year's “Poor People's Energy Outlook” report, published by the NGO *Practical Action*, energy poverty has left more than 1 billion people in developing countries without access to adequate healthcare. Medical staff is forced to treat emergency patients in the dark, and health centres lack the power to store vaccines or sterilise medical supplies. In India alone, nearly half of all health facilities – serving almost 600 million people – lack electricity, with a further 255 million people being served by health centres without electricity in sub–Saharan Africa, where more than 30% lack power. Even where health centres do have access to power, frequent power shortages significantly hamper the ability to provide quality care. In Kenya, only 25% of facilities have a reliable energy supply, and blackouts happen at least six times a month, for an average of 4.5 hours at a time, affecting services and leading to wasted vaccines, blood and medicines that require constant storage temperatures. (*The Guardian*, 7 Mar 2013)

• Galvanized by the “Sustainable Energy for All” initiative, led by UN secretary general Ban Ki–moon, energy has shot up the international agenda. The initiative aims to achieve universal access to energy by 2030, along with efficiency gains and increased use of renewable energy. Non–governmental organizations, however, warn that too little attention has been paid to the needs of critical community services, putting progress on development goals, particularly on health and education, at risk. (*The Guardian*, 7 Mar 2013)

• According to a report by the Pew Charitable Trusts, China overtook the United States as the global leader in clean energy investment in 2012, while American spending on renewable energy sources dropped nearly 40%. The report concluded that the center of gravity in the clean energy has shifted from the United States and Europe to China. China’s leaders are intensely focused on clean energy, with aggressive targets that helped the rapid growth of the country’s solar and wind industries. China attracted US$ 65 billion in clean energy investment in 2012, which is 30% of all renewable investment in the G20 economies. China is installing solar energy partly because the western European market for its solar products is drying up. China is also a major manufacturer of wind turbines. (*The Modesto Bee*, 6 Jun 2013)

• MarketResearchReports.Biz announced its new report on “Thermoelectric Energy Harvesting 2013–2023: Devices, Applications, Opportunities”. Thermoelectric generators are devices that convert temperature differences into electrical energy. The underlying principle phenomenon is known as the Seebeck effect: the conversion of a temperature differential into electricity at the junction of two materials. Although thermoelectric phenomena have been used for heating and cooling applications in the past, electricity generation has only seen limited market, but in recent years that interest has increased. (*WatchList News*, 8 Jun 2013)

• Chilean businesses seeking to curb their carbon emissions should be able to tap into 600 MW of new, green energy projects. This is due to a new, US$ 1.4 billion joint venture between Mainstream Renewable Power and the investor company Actis. The Ireland–based renewable energy developer confirmed plans to bring 450 MW of wind energy projects and 150 MW of solar power capacity into operation, or at least construction, in Chile by early 2016. Under the terms of the deal, Actis will own 60% of the joint venture while Mainstream will own the remaining 40%. Mainstream will develop all the projects and manage them once they are completed, with the joint venture company investing at the point of financial close for each scheme. In a statement, Mr Eddie O'Connor, Mainstream's chief executive, said the new projects were “...primarily aimed at helping to curb emissions from large industrial businesses in the country”. (*Business Week*, 10 Jun 2013)

## ENVIRONMENT

• The year 2012 was among the 10 warmest years on record, as reported by agencies such as National Oceanic and Atmospheric Administration (NOAA) and NASA. The global temperatures in 2012 were higher than the long–term average for the 36^th^ consecutive year. It appears that the average global temperature has risen about 1.4F since 1880. NASA declared the year 2012 as the 9^th^ hottest year on record, while NOAA ranked it 10^th^ on record. (*The Guardian*, 16 Jan 2013)

• In his inaugural address, the US President Mr Barack Obama made climate change a second–term priority. It is now emerging that he could bypass Congress to implement much of his environmental agenda unilaterally. He could revive the failed 2009 cap–and–trade legislation through regulation, with legal authority derived from the four–decade–old Clean Air Act, and a 2007 Supreme Court decision applying it to carbon emissions. This should allow imposing curbs on coal–fired electric plants and limit methane emissions from hydraulic fracturing, according to environmentalists' sources. (*Bloomberg*, 23 Jan 2013)

• A poll was conducted among 200 000 people to propose six life–changing priorities for their local environments to help planning post–2015 global agenda. One of the top three priorities in the world as a whole, for both men and women, for people of all ages, and in all types of countries was “an honest and responsive government”. The other two priorities consistently in the top three for most groups were a good education and better healthcare. (*The Guardian*, 25 Mar 2013)

• The Hunger–Nutrition–Climate Justice (HNCJ) conference, held in Dublin, Ireland, considered the issue of joint effects of climate change and hunger on the poor nations from the perspective of “climate justice”. This is an approach to climate change focusing on the rights of vulnerable people who are the least responsible for causing climate change, but among the most affected. (*IRIN,* 24 Apr 2013)

• Most of the attention on global environmental issues goes to climate change, and the carbon dioxide we’re adding to the atmosphere may already be changing the climate for the worse. However, according to researchers from New York’s Mount Sinai Medical School and the Blacksmith Institute, a NGO that focuses on industrial pollution in the developing world, high lead exposure in small children may be an even more pressing immediate issue. It has been linked to many complications later in life, including lower IQ, hyperactivity, behavioural problems and learning disabilities. This is a disorder of the poor, who live in the crowded urban tenements and near toxic industrial sites. In the postwar era, lead contamination was common even in wealthy countries because of the widespread use of leaded gasoline, paints and soil; when it was removed in the 1970s, lead contamination plummeted, with blood lead levels among pre–school children reducing from 16.5 micrograms/dl in 1978 to just 3.6 in 1993. Getting lead out of the environment might have been one of the most important public–health actions the US has ever taken. (*TIME*, 8 May 2013)

## FOOD, WATER AND SANITATION

• The UK Institute of Mechanical Engineering’s (IMechE) report on food waste estimated that 30–50% of food produced globally is wasted. In less developed countries this waste is largely due to poor practices in harvesting, storage and transportation. In developed countries the wastage mainly occurs further along the line of food production and consumption, due to behaviour of supermarkets and consumers. In the UK, for example, up to 30% of the vegetable crop is rejected because it does not conform to the narrow range of physical characteristics required by the major supermarkets, with further 30–50% of food discarded by the purchaser. The report stated that eliminating the waste could provide 60–100% more food for consumption. To help prevent a global food crisis, the IMechE report recommended that engineers should be involved in improving food production in developing countries, while rapidly developing countries should incorporate ‘waste minimisation thinking’ into their planning of transport and storage facilities, and high–income countries should work on changing consumer behaviour. (*The Guardian*, 07 Jan 2013)

• More than 150 organisations and charities have banded together to create the “*enough food for everyone IF*” campaign, which aims to lobby MPs and bring hunger to the world agenda at the G8 summit. The campaign is based on the principle that the world can produce enough food to sustain its population if the richest nations tackle the “four big IFs”: (i) provide enough aid to help families feed themselves and prevent children dying from hunger; (ii) stop big companies from tax–dodging in developing countries; (iii) prevent poor farmers from being forced out of their land, and allow them to grow food, not fuel; and (iv) governments and big companies become transparent about their actions which act as barriers to people getting enough food. This is the largest collaboration in the charity sector since the 2005 “*Make Poverty History*” campaign. It focuses on the responsibility and accountability of governments and international corporations. Critics have reflected that the campaign is too similar to the “*Make Poverty History*” campaign and risks losing public interest by the apparent lack of change since 2005. (*BBC News*, 23 Jan 2013)

• At the current rate of progress, the world is set to miss the global sanitation target for 2015 by over half a billion people. More people today have access to a cell phone than to a clean toilet. While the drinking water global target was met last year, nearly a billion people still lack access to an improved drinking water source. The World Bank estimated that economic cost of the water and sanitation crisis result in economic losses estimated at US$ 260 billion annually in low– and middle–income countries, which is about 1.5% of their cumulative GDP. (*The World Bank / The Water Blog*, 11 Feb 2013)

• The Hunger–Nutrition–Climate Justice (HNCJ) conference, held in Dublin, Ireland, was organized by Irish Aid, the Mary Robinson Foundation, CGIAR and the World Food Programme (WFP). Studies conducted in Ethiopia, India, Kenya and Niger showed that children born during natural hazards, like droughts or floods, are more likely to be malnourished, while poor countries, which are already struggling with hunger and food insecurity, are increasingly likely to face these natural hazards. “Joined–up approach” – also known as the “nexus” approach – seeks to find solutions for such circular problems based on the interconnections between various sectors or disciplines. For instance, addressing interconnected malnutrition and climate change problems would involve working across health, agriculture, environment, water and land management sectors. (*IRIN,* 24 Apr 2013)

• Researchers from the Potsdam Institute for Climate Impact Research, Germany, calculated the food growing capacity of every country in the world and compared it with food requirements in the present, and projected forward to 2050. Their model employed climate data, soil type and land–use patterns, population projections and food and water consumption. Although many countries choose to import food right now, the model showed that there are surprisingly few – 66 countries, or 16% of the world's population – that could not maintain the same diet and still be food self–sufficient. The countries with the most reliance on imports were found in North Africa, the Middle East and Central America. However, over half of the world's population could depend on imported food by 2050. Potsdam Institute projection suggests population growth would increase imported food, even without climate change, because they are limited by lack of land or water. (*The Guardian*, 7 May 2013)

## PEACE AND HUMAN RIGHTS

• According to a large international study of national defence ministries and armed forces, most countries lack the tools to prevent corruption in the arms trade. Transparency International's (TI) report showed that a number of most lucrative potential markets for arms, including Saudi Arabia, Indonesia and Oman, are also among countries with “a very high risk” of corruption. TI's Government Defence Anti–Corruption Index analyses measures by 82 countries to reduce corruption risks. (*The Guardian*, 29 Jan 2013)

• A new UN report estimated that millions of people are still being trafficked for sexual exploitation and forced labour. The encountered victims were from at least 136 different nationalities, and they have been detected in 118 countries. The majority of victims are women, although the number of children is also increasing. (*Miami Herald*, 12 Feb 2013)

• United Nations officials joined millions of people globally who took a stance against violence against women. This public action was a part of the “One Billion Rising” campaign – sponsored by the V–day Organization – which seeks to mobilize men and women around the world on Valentine’s Day. The aim of the campaign is to stop violence against women and girls, including rape, battery, incest, female genital mutilation (FGM), and sex slavery. (*UN News*, 14 Feb 2013)

• In an excellent analysis, Ms Mary Robinson, Mr Kevin Rudd and Ms Judy Cheng–Hopkins suggest that peace, security, good governance and the rule of law must be included in the new Millennium Development Goals. They remind that, in September 2000, world leaders unanimously adopted the Millennium Development Goals (MDGs) – a series of specific targets for poverty eradication, universal primary school enrolment, gender equality, reduction in child and maternal mortality, combating major disease and ensuring environmental sustainability. The MDGs encouraged developing countries and the rest of international community to take concrete and tangible steps towards achieving the set targets, with a notable progress along the way. However, a sobering fact is that no fragile or conflict–affected low–income country has managed to achieve a single MDG. (*The Huffington Post*, 12 Mar 2013)

• The newly elected Pope Francis declared that he “...would like to see a church that is poor and is for the poor”. The first Latin American and the first Jesuit pontiff explained to the media that he chose to name himself after St Francis of Assisi, because the saint devoted his life to peace and the poor. In a clear signal of his desire to reset the priorities of the embattled Catholic Church after Benedict XVI's intellectual, remote–seeming reign, Francis added that the reminder had made him think of St Francis – a man “who wanted a poor church”. Only 16% of the world's population is presently Catholic, but some 42% are Latin American and 15% African. (*The Guardian*, 16 Mar 2013)

## SCIENCE AND TECHNOLOGY

• Mobile phone technology is frequently proposed as an attractive solution to address health challenges in low resource settings. However, two recent reviews – on the impact of mHealth on behaviour change and service delivery – didn't manage to find proper evidence that mHealth really works in a desired way. Only three of 75 trials that assessed mHealth's impact on health behaviour change or improved disease management were conducted in low– or middle–income countries. Further high quality trials are required to establish the true potential and effectiveness. (*SciDev.Net*, 15 Jan 2013)

• The first major trial of a new booster vaccine against tuberculosis (TB) since Bacillus Calmette–Guerin (BCG) was introduced in 1921 has failed. BCG is only partially effective against the mycobacterium that causes TB and there are several ongoing efforts to introduce new vaccines. The latest, MVA85A, failed to protect babies who already received BCG. The trial was conducted in South Africa and involved 2794 healthy children aged four to six months, half of whom were vaccinated and then followed up for an average of two years. The researchers found 32 cases of TB in those who had received the vaccine and 39 in the placebo group, i.e. not a significant result. (*BBC*, 4 Feb 2013)

• The intrinsic value of the “Human Genome Project”, completed in 2003 with mainly public resources, has since been circumscribed by the expansion of intellectual property rights over genomic DNA. An estimated 20% of the human genome is now subject to patents that can impose complex legal and cost constraints on medicines and diagnostics. The key issue is not an argument against patenting of inventions based on “modified” gene sequences, but rather the practice that enables the isolated, but otherwise unmodified, genomic DNA to be included in patent claims. This intellectual property practice is not universal, and countries such as Brazil do not allow this practice, but its intellectual property framework is nevertheless consistent with World Trade Organization intellectual property obligations. In January, the European Parliament questioned whether those responsible for important policy matters understood the consequences for the developing world of allowing intellectual property rights over unique products of nature, such as the human genome. The 2002 “Genomics and World Health” report of the World Health Organization's advisory committee on health research highlighted problems with patents locking away the use an original gene sequence, with the follow–up initiative in 2010 suggesting options to deal with a growing “genome divide”. However, until recently little scrutiny has been applied to legal and policy frameworks that enable the incremental expansion of intellectual property rights into this scientifically important area of health. (*The Guardian*, 12 Mar 2013)

• The Canada Gairdner International Awards honoured three areas of research in 2013: Drs Harvey J. Alter and Daniel W. Bradley received the award for their contributions to the discovery and isolation of the hepatitis C virus, with Dr Michael Houghton also selected, but declining to receive the award. Dr Stephen Joseph Elledge was recognized for his work in DNA repair, while Sir Gregory Winter was honoured for creating synthetic human antibodies. Dr King K. Holmes was selected for the Canada Gairdner Global Health Award for his work on defining and treating HIV and other sexually transmitted diseases, and The Canada Gairdner Wightman Award went to Dr James C. Hogg for his research and leadership in the field of chronic respiratory diseases. (*Gairdner Foundation*, 20 Mar 2013)

• Scientists studying tuberculosis (TB) patients in Tamil Nadu, India, have found that a quarter were diabetes sufferers. The connection between the two diseases that has already been recognised in industrialised countries, and is emerging even more strongly in regions such as Asia and Africa. According to Dr Anil Kapur, managing director of the World Diabetes Foundation, which funded the project, “...people with diabetes in the developing world are two or three times more likely to contract TB, compared with those without diabetes”. Moreover, having diabetes seems to reduce response to TB treatment, so that one disease reinforces the adverse effects of the other. (*Financial Times*, 21 Mar 2013)

**Contributors:**

Rachel Banfield, Frances Barclay, Vijna Hiteshna Boodhoo, Katherine Booth, Rachel Burge, Michael Charteris, Alexander Fullbrook, Jenny Hall, Ewan D. Kennedy, Nethmee S. Mallawaarachchi, Victoria Stanford, Goran Zangana

